# Supplementary material for: Divergent evolution of male-determining loci on proto-Y chromosomes of the housefly
Source: Nat Commun. 2024 Jul 16;15:5984. doi: 10.1038/s41467-024-50390-1 (PMC11252125; doi:10.1038/s41467-024-50390-1)
Supplement: Supplementary file 1 — Supplementary information [file 41467_2024_50390_MOESM1_ESM.pdf]

Supplementary Table 1 Overview statistics of the M3, M5 and *aabys*-male genomes in comparison with published *aabys* genome

Genome	*aabys*	*aabys*-male	M3	M5
**Total length (bp)**	750,403,944	893,747,508	1,318,800,909	1,333,442,172
**Contig number**	20,487	18,213	11,176	4,327
**Largest Contig/Scaffold**	2,348,962	454,643	12,933,002	37,055,696
**Contig number (>= 10000 bp)**	6,714	16,975	10,169	10195
**GC (%)**	35.11	35.99	35.16	34.78
**N50**	226,573	63,778	617,562	7,800,370
**N75**	82,880	40,079	172,126	1,980,037
**L50**	809	4,206	471	47
**L75**	2,176	8,651	1,468	134
**BUSCO score***	C:96.5% (3172)<br>S:95.5% (3138)<br>D:1.0% (34)<br>F:1.7% (56)<br>M:1.8% (57)	C:80.4% (2642)<br>S:79.1% (2600)<br>D:1.3% (42)<br>F:4.8% (158)<br>M:14.8% (485)	C:98.9% (3249)<br>S:58.2% (1911)<br>D:40.7% (1338)<br>F:0.5% (17)<br>M:0.6% (19)	C:99.2% (3258)<br>S:69.8% (2292)<br>D:29.4% (966)<br>F:0.3% (10)<br>M:0.5% (17)

* Total BUSCO groups searched: 3285 (Universal protein-coding genes in dipteran lineages). C: Complete BUSCOs; S: Complete and single-copy BUSCOs; D: Complete and duplicated BUSCOs; F: Fragmented BUSCOs; M: Missing BUSCOs.

Supplementary Table 2 BLAST results of aligning *Mdmd* and *Mdncm* ORF sequences to the 4 contigs in the M3 genome, 2 contigs in the M5 genome and 5 contigs in the *aabys*-male genome that contain *Mdmd*-like sequences

Subject	Query	Genome	Subject length (bp)	Max score	Total score	E-value	Identity (%)
Mdmd (ORF)	Contig6762	M3	3759	3077	39190	0	97.05
	Contig7871		3759	3544	1.537e+05	0	100
	Contig624		3759	939	1682	0	85.42
	Contig5307		3759	961	1715	0	85.86
	tig00004758	M5	3759	3517	13804	0	99.74
	tig00000533		3759	939	1699	0	85.42
	contig_6317_pilon	*aabys*-male	3759	483	2215	8.00E-138	88.75
	contig_2268_pilon		3759	3099	11939	0	97.27
	contig_2269_pilon		3759	2787	18072	0	96.37
	contig_12930_pilon		3759	3506	8652	0	99.64
	contig_12873_pilon		3759	760	760	0	83.43
Mdncm (ORF)	Contig6762	M3	3510	749	4261	0	83.07
	Contig7871		3510	749	19542	0	83.07
	Contig624		3510	3363	6035	0	97.89
	Contig5307		3510	3469	6074	0	98.87
	tig00004758	M5	3510	743	1486	0	82.95
	tig00000533		3510	3469	6212	0	98.87
	contig_6317_pilon	*aabys*-male	3510	No significant similarity found			
	contig_2268_pilon		3510	754	1437	0	83.19
	contig_2269_pilon		3510	747	2484	0	83.09
	contig_12930_pilon		3510	686	686	0	81.77
	contig_12873_pilon		3510	3533	6664	0	99.48

Supplementary Table 3 BLAST results of matches for partial replicates of predicted genes in the M^{III}-contigs and against M3 genome and with the best match

Description	Sequence length (bp)	Accession No.	Contig No.	Max score	Total score	Query cover	E value	Per. ident
ribose-phosphate pyrophosphokinase 1	2272	XM_005183825.3	M^{III}-contig-1	985	3226	41%	0	93.87%
			2374	1375	4020	94%	0	99.60%
cytochrome P450 6D3 (CYP6D3)	14009	AF200191.1	M^{III}-contig-1	723	8159	13%	0	86.33%
			M^{III}-contig-2	643	8372	11%	0	87.19%
			7484	3722	1.19E+05	89%	0	98.49%
Musca domestica 60S ribosomal protein L22-like	595	XM_011296757.1	M^{III}-contig-1	560	844	93%	2.00E-161	96.47%
			M^{III}-contig-2	440	779	53%	5.00E-125	91.56%
			4724	1074	1074	100%	0	99.16%
Musca domestica putative protein TPRXL	790	XM_005189509.1	M^{III}-contig-1	507	1751	100%	3.00E-145	88.14%
			M^{III}-contig-2	368	654	31%	3.00E-103	93.52%
			9512	782	1711	100%	0	100.00%
alpha-1,3-mannosyl-glycoprotein 4-beta-N-acetylglucosaminyltransferase A	6282	XM_020038408.1	M^{III}-contig-1	394	497	7%	2.00E-110	84.50%
			M^{III}-contig-2	442	1740	7%	2.00E-124	84.28%
			2611	3238	18076	99%	0	93.15%
putative mediator of RNA polymerase II transcription subunit 26	12199	XM_020039898.1	M^{III}-contig-1	361	498	3%	5.00E-100	86.31%
			37	13732	25446	99%	0	96.88%
DNA topoisomerase I, mitochondrial-like	895	XM_020039943.1	M^{III}-contig-1	283	2906	63%	7.00E-78	89.95%
			2844	1568	1795	100%	0	98.33%
defensin-1 mRNA	1012	KC920907.1	M^{III}-contig-1	141	266	13%	5.00E-35	86.57%
			3896	1683	1979	97%	0	97.38%
protein sax-3	6577	XM_020036366.1	M^{III}-contig-1	137	229	1%	4.00E-33	89.72%
			960	3650	10509	92%	0	97.15%
putative uncharacterized transmembrane protein DDB_G0293652	1677	XM_020039173.1	M^{III}-contig-2	1229	4488	100%	0	93.50%
			619	3009	4064	100%	0	99.05%
Transcription elongation factor SPT6-like	3405	XM_020034885.1	M^{III}-contig-2	1171	7704	54%	0	92.24%
			3766	4063	19462	100%	0	97.39%
ATP-dependent Clp protease proteolytic subunit, mitochondrial	1565	XM_005189536.3	M^{III}-contig-2	913	2190	48%	0	93.10%
			6518	2658	9303	100%	0	99.86%
transposon MdmaT1a nonfunctional transposase	961	AF315724.1	M^{III}-contig-2	869	869	84%	0	85.94%
			10355	1099	37845	91%	0	91.05%
ABC membrane transporter (white)	2180	AY055821.1	M^{III}-contig-2	496	496	18%	4.00E-141	89.57%
			1321	1094	66494	100%	0	83.43%
protein prickle	7369	XM_020035464.1	M^{III}-contig-2	350	700	2%	1.00E-96	97.56%
			5687	3980	12146	95%	0	97.91%
clock protein period	6573	AH007818.2	M^{III}-contig-2	344	569	8%	5.00E-95	82.11%
			1194	8026	86233	93%	0	97.61%
partial bcd gene for bicoid protein	1293	AJ297850.1	M^{III}-contig-2	185	185	36%	6.00E-48	74.40%
			10701	1434	3587	99%	0	97.95%

Supplementary Table 4 Overview of the repeat sequences in the M^{III}-contigs

Contig	Class	Type	Num	Sum
M^{III}-contig-1	Class I Retrotransposon	LINE/CR1	1	94
		LINE/I-Jockey	9	
		LINE/R1-LOA	19	
		LINE/RTE-BovB	1	
		LTR/Copia	3	
		LTR/Gypsy	26	
		LTR/Pao	35	
	Class II DNA transposons	DNA/CMC-EnSpm	3	42
		DNA/hAT-Blackjack	3	
		DNA/TcMar-Tc1	11	
		RC/Helitron	25	
		Low_complexity	4	
		Simple_repeat	43	
		Unknown	145	
M^{III} -contig-2	Class I Retrotransposon	LINE/CR1	24	141
		LINE/I-Jockey	12	
		LINE/Penelope	20	
		LINE/R1-LOA	48	
		LINE/RTE-BovB	2	
		LTR/Gypsy	23	
		LTR/Pao	12	
	Class II DNA transposons	DNA/CMC-EnSpm	1	55
		DNA/TcMar-Tc1	17	
		RC/Helitron	37	
		Low_complexity	9	
		Simple_repeat	42	
		Unknown	297	

Supplementary Table 5 Positions where SNPs are found in intact *Mdmd* gene from M^{II}, M^{III}, M^{V}, and M^{Y}

Position	310	512	527	1020	1184	1596	1712	1722	1765	1853	1997	2040	2671	2683	2942	3093	3257	3272	3282	3425	Total
M^{II}	A	C	A	G	C	T	G	T	A	G	T	G	A	A	G	C	G	C	T	T	4
M^{III}	A	A	A	A	C	C	A	C	G	T	C	G	A	T	A	T	G	C	C	T	8
M^{V}A	A	A	G	G	C	T	A	T	A	G	T	G	A	A	G	T	G	C	C	T	1
M^{V}B	A	A	G	G	C	T	A	T	A	G	T	A	A	A	G	T	G	C	C	T	2
M^{Y}	G	A	A	G	A	T	A	T	A	G	T	G	G	A	G	T	A	G	C	C	6
Consensus	A	A	A	G	C	T	A	T	A	G	T	G	A	A	G	T	G	C	C	T	

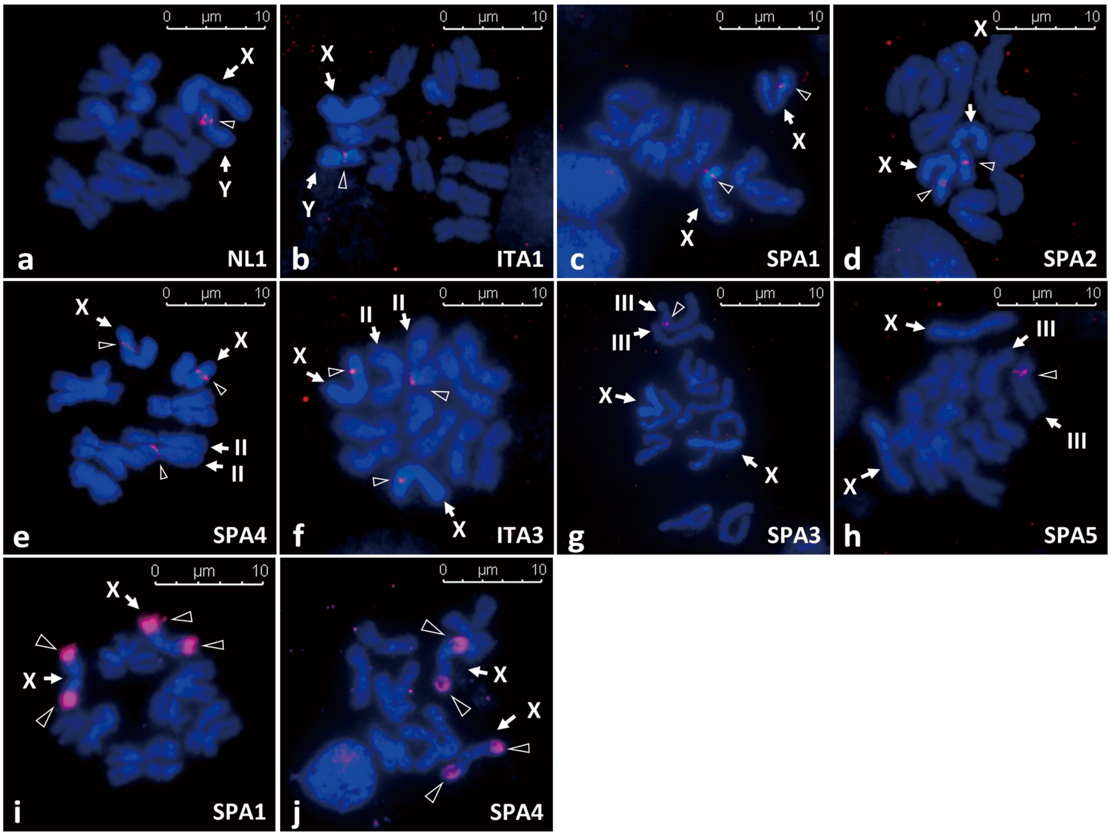

Supplementary Fig. 1 FISH localization using *Mdmd*-specific probe and Mix probe in samples from various geographic strains. *Mdmd*-specific probe resulted in localization of *M* loci on the Y chromosome (**a, b**), the X chromosome (**c, d, e, f**), chromosome II (**e, f**) and chromosome III (**g, h**). M^{Y}, M^{II} and M^{III} all showed pericentromeric location on the short arms of each chromosome. M^{X} was located in the middle of one arm of the X chromosome. Particularly, homozygous M^{X} are observed because of the existence of tra^{D}, a dominant variant of *transformer* gene. tra^{D} directs female development regardless of the presence of *M*, and thus, allows both males and females to carry *M* (for details see ref[20]). Mix probe was applied to the SPA1 (**i**) and SPA4 (**j**) samples. The results show co-localization of MAS repeats and M^{X}. The M^{X} signals are inseparable from the signals of MAS repeats signals indicating the adjacency of the two. Positive signals are shown in red and indicated by open triangles, chromosomes are indicated by arrows. Mitotic chromosomes are shown in blue. Signals were only considered as a successful hybridization if they were observed with consistent chromosomal locations on at least 20 metaphase nuclei on each slide. For each strain, 2-3 individuals were tested to ensure reproducibility.

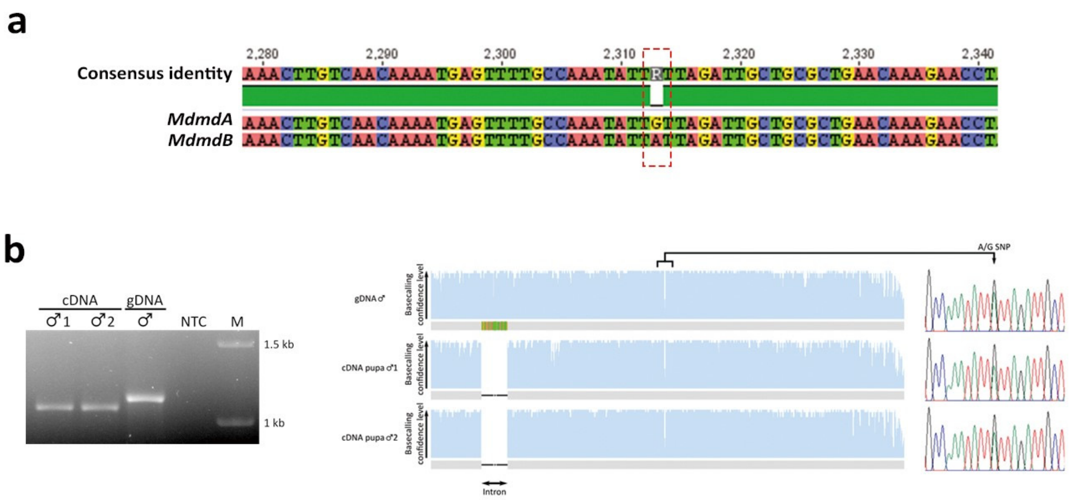

Supplementary Fig. 2 Genomic sequence alignment and transcripts of the *Mdmd* copies in M^V. **a:** Only one nucleotide substitution (indicated by the dashed line box) is found between the two *Mdmd* copies. **b:** PCR amplification of *Mdmd* fragments using cDNA of two M5 male samples. The amplified fragments from the cDNA are shorter than that of the gDNA as the *Mdmd* transcripts do not contain the intron. NTC: control. M: ladder. Upon Sanger sequencing, base-calling sequencing electropherogram shows that in both gDNA and cDNA fragments, a double peak representing G and A exist at the SNP location. This demonstrates that both *MdmdA* and *MdmdB* transcribe.

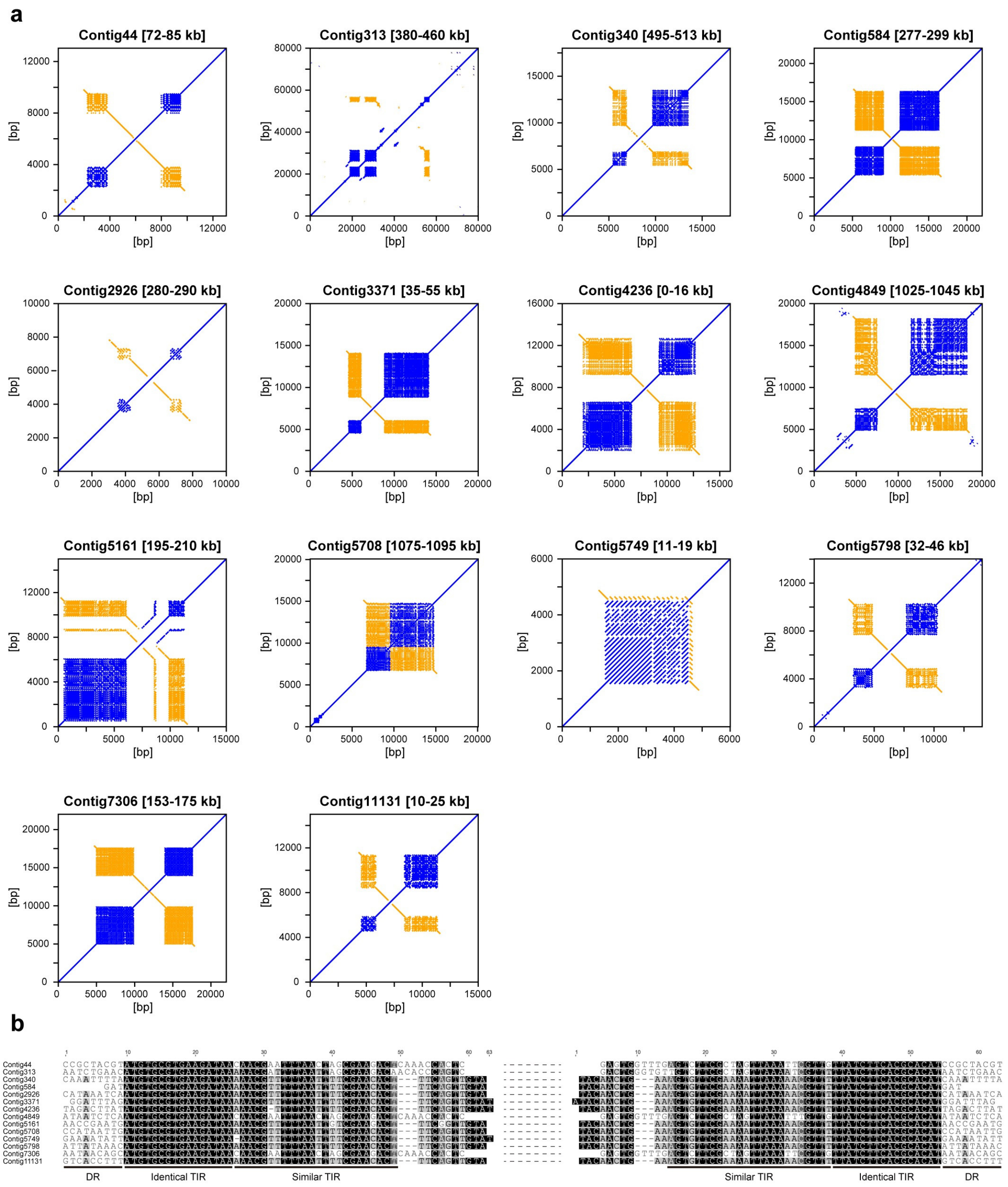

Supplementary Fig. 3 Examples of genomic regions that show similar structures to M^V and contain identical Terminal Inverted Repeats (TIR). **a:** Self-alignment visualizations of 14 genomic regions containing the same interspersed tandem repeat blocks and TIRs as M^V. M^V-like palindromic structures are observed in all of them. The matched sequences are shown in blue (with the same orientation) and orange (with the reverse orientation). The wordsize used for similarity search was set to 50. **b:** All examined palindromic regions contain identical TIRs. Flanking those TIRs are direct repeats (DRs) that are mostly 9 bp (12 out of 14 examined regions) but differ in sequence contents.

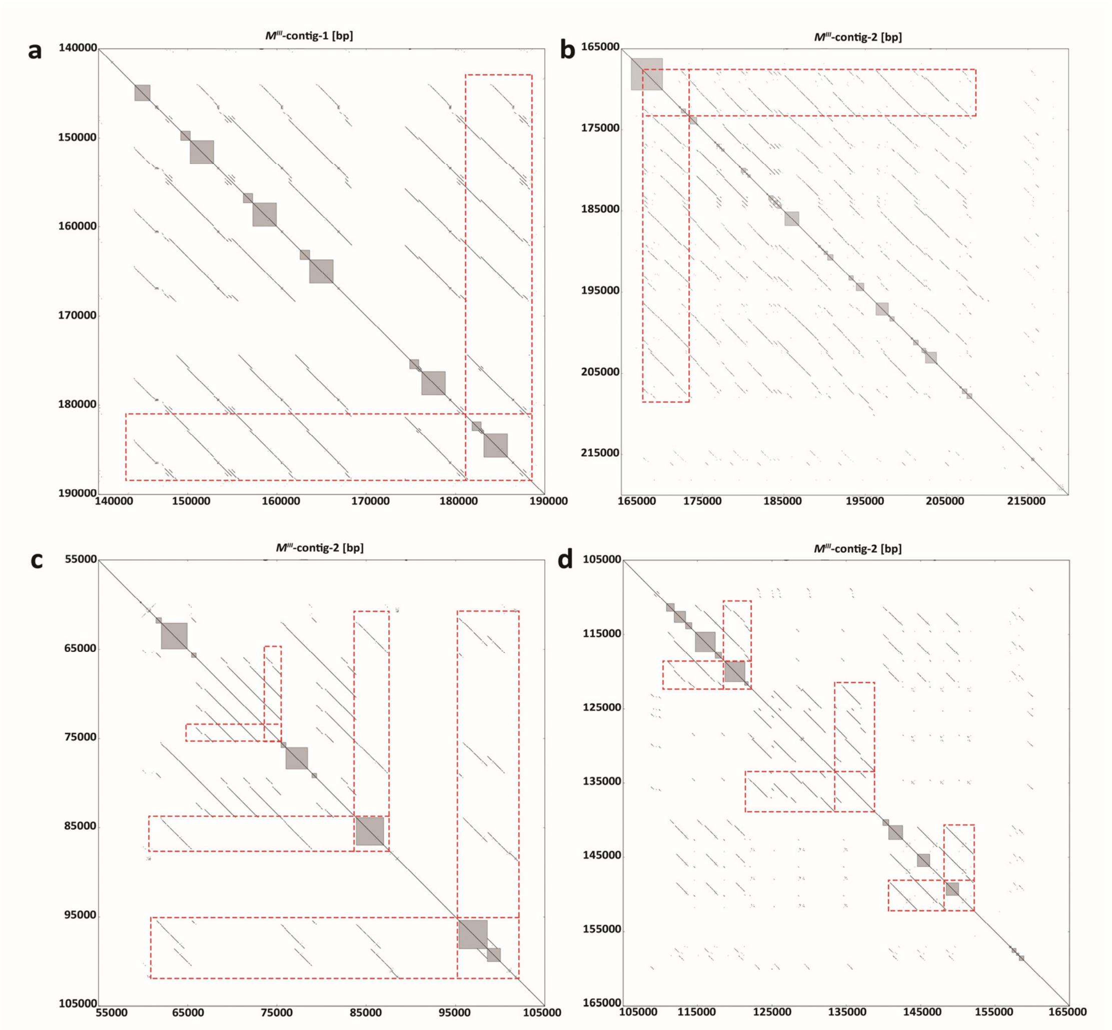

Supplementary Fig. 4 Self-alignment visualization of 4 examples of duplicated regions on the M^{III}-contigs. The grey squares represent the *Mdmd* sequences. The red dashed line boxes indicate the duplicated unit. The duplicated unit vary in length and range as it can be *Mdmd* with its upstream and downstream sequences (**a**), partial *Mdmd* sequences with its downstream sequences (**b**), or only *Mdmd* flanking sequences (**c**, **d**). The wordsize used for similarity search was set to 25.

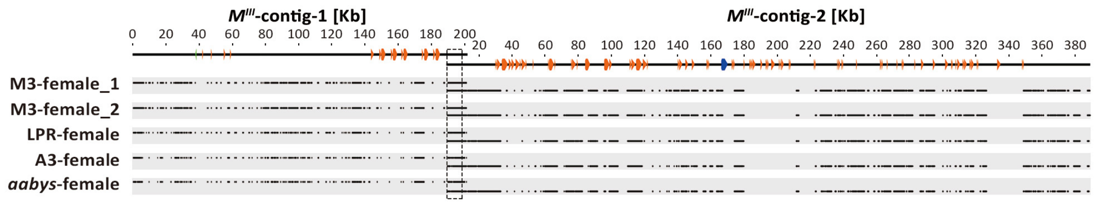

Supplementary Fig. 5 The coverage in five female genomic datasets of the M^{III}-contigs. The similarity to the M^{III}-contigs for each dataset is represented by a horizontal line consisting of individual dots that indicate sufficient coverage. The lack of similarity is visible by the absence of dots and thus a discontinuous line. Unmapped parts indicate M^{III}-specific sequences, which disperse over the M^{III}-contigs. All five replicates showed comparable coverage patterns. The schematic drawing of M^{III}-contigs on the top show the distribution of *Mdmd* sequences, wherein the fragmented replicates are represented by orange (sense order) or green (antisense order) triangles. The complete *Mdmd* genes are represented by blue triangles. The dashed line box indicates the part where the two M^{III}-contigs share high homology.

Supplementary Discussion

Identification of DSB/homologous repair traces in M^{III} tandem duplications

Fiston-Lavier *et al.* (2007) described a particular case of tandem duplication formation via re-invasion process during double-strand break/homologous repair mechanism. Several sequence traces are expected to exist as a result. For instance: 1. The pairwise alignment of the template sequence and the sequences with the tandem duplication would exhibit a gap on the template. 2. Repeats are strictly in tandem within the newly synthesized sequence. 3. Homologous fragments should be detected between the template and each copy of the duplication. We examined a duplicated region on one M^{III}-contigs to see if we could find such traces. We focused on the 170-190 kb region on M^{III}-contig-2 as this is M^{III}-specific and is considered to have recently originated after the translocation from the Y to autosome III. Sequence traces are thus less likely lost because of degeneration.

We found one region at approximately 183-185 kb on M^{III}-contig-2 (duplicated sequence, referred to as DS) representing replication of the sequence at approximately 170-173.5 kb (template sequence, referred to as TS). Unlike the TS, the DS contains one fragment that replicated twice, thereby resulting in a total of three tandem copies (fig. S6). By examining pairwise alignment between TS and DS, microhomology traces were observed in both TS and DS. One trace directly flanked the gap in TS, whereas two are located at the end of additional duplications in DS (indicated by red boxes, fig. S6). Because of observed traces, we inferred that double-strand break/homologous repair mechanism caused examined tandem replications.

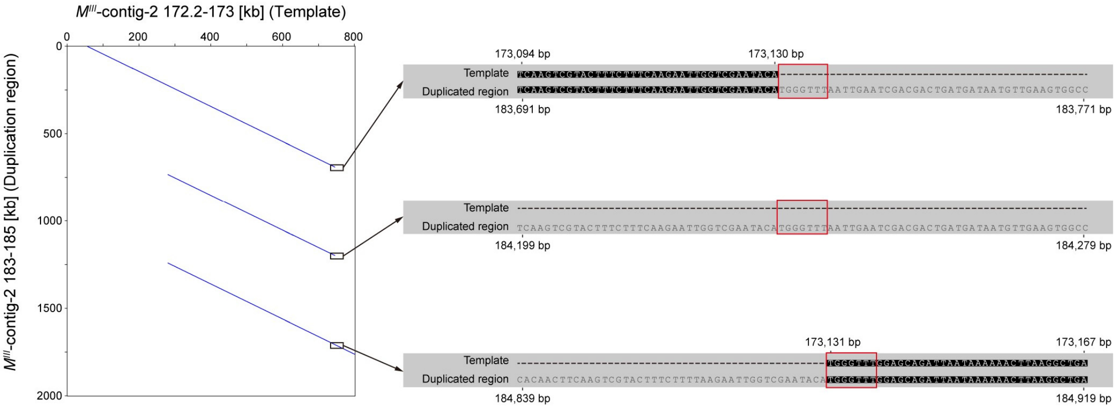

Supplementary Fig. 6 Alignment of a tandemly duplicated region in comparison to its template sequence on M^{III}-contig-2. Dotplot visualization demonstrates (on the left) two additional tandem copies in the duplicated region. Paiwise alignment between the template and the duiplicated region reveals microhomology traces (indicated by red box), that allows dissociation and upstreams re-invasion.

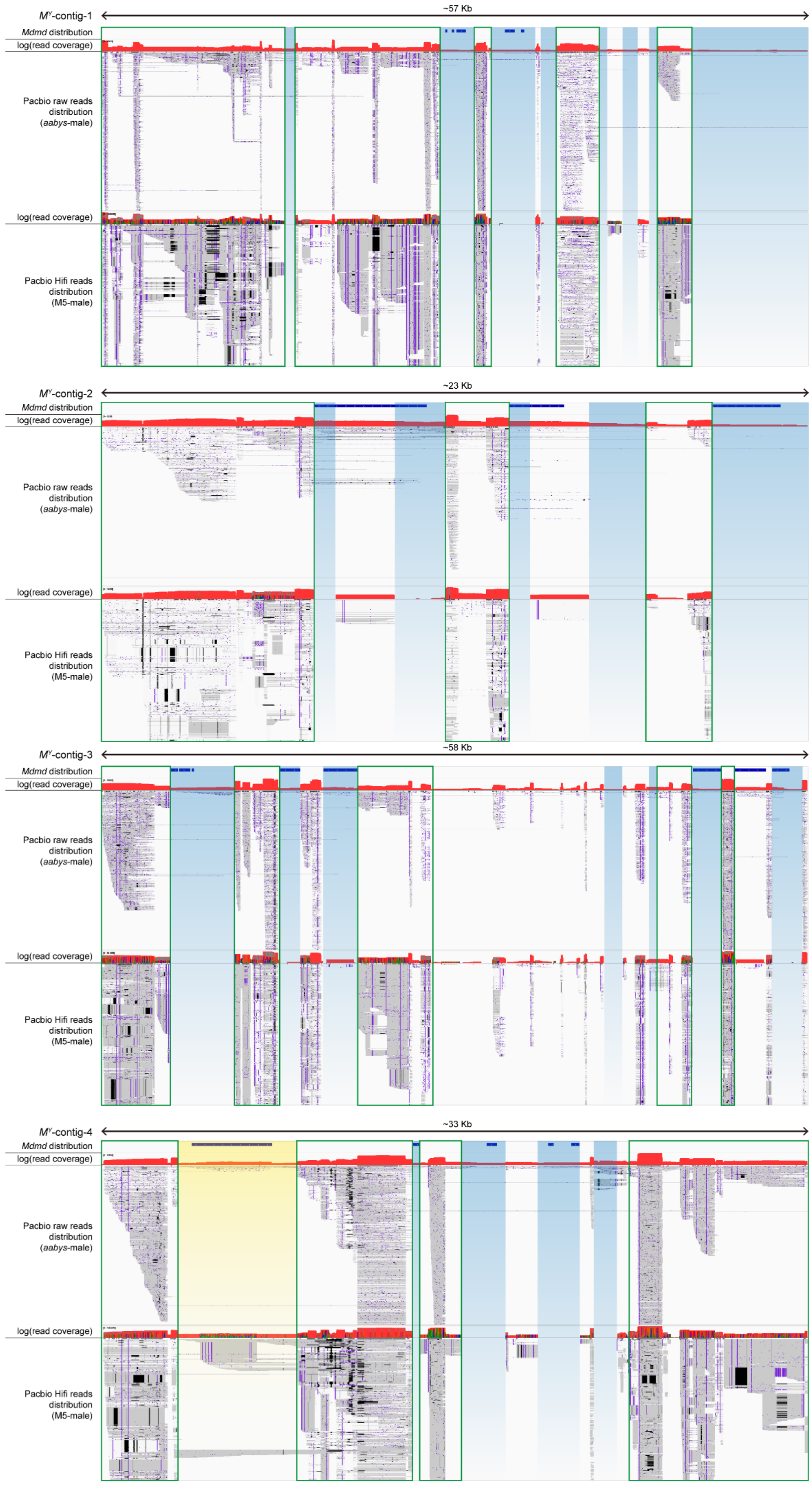
M^Y-contig-1
~57 Kb
Mdmd distribution
log(read coverage)
Pacbio raw reads distribution (*aabys*-male)
log(read coverage)
Pacbio Hifi reads distribution (M5-male)
M^Y-contig-2
~23 Kb
Mdmd distribution
log(read coverage)
Pacbio raw reads distribution (*aabys*-male)
log(read coverage)
Pacbio Hifi reads distribution (M5-male)
M^Y-contig-3
~58 Kb
Mdmd distribution
log(read coverage)
Pacbio raw reads distribution (*aabys*-male)
log(read coverage)
Pacbio Hifi reads distribution (M5-male)
M^Y-contig-4
~33 Kb
Mdmd distribution
log(read coverage)
Pacbio raw reads distribution (*aabys*-male)
log(read coverage)
Pacbio Hifi reads distribution (M5-male)

Supplementary Fig. 7 Mapping Pacbio raw reads of *aabys*-male (M^Y-containing) and M5-male (M^V-containing) to M^Y-contigs. Distribution of *Mdmd* sequences is showed by dark blue blocks in the first track in each figure. Blue shading indicates major M^Y-specific regions, which only showed coverage in *aabys*-male reads, but not in M5-male reads. Yellow shading indicates the region containing the intact *Mdmd* copy. As M^Y and M^V share homology for this region, both *aabys*-male reads and M5-male reads show coverages. Green boxes indicate major regions that show higher coverages than M^Y-specific regions and are mapped not only by *aabys*-male reads but also by M5-male reads. As M^V and M^Y-loci do not share homology for those regions, mapped M5-male reads are likely non-*M*-related sequences, which are potentially homologous sequences on the X chromosome.
