# Supplementary material for: Divergent evolution of male-determining loci on proto-Y chromosomes of the housefly
Source: Nat Commun. 2024 Jul 16;15:5984. doi: 10.1038/s41467-024-50390-1 (PMC11252125; doi:10.1038/s41467-024-50390-1)
Supplement: Supplementary file 3 — Reporting Summary [file 41467_2024_50390_MOESM3_ESM.pdf]

nature portfolio

Corresponding author(s): Xuan Li

Last updated by author(s): Jun 19, 2024

Reporting Summary

Nature Portfolio wishes to improve the reproducibility of the work that we publish. This form provides structure for consistency and transparency in reporting. For further information on Nature Portfolio policies, see our Editorial Policies and the Editorial Policy Checklist.

Statistics

For all statistical analyses, confirm that the following items are present in the figure legend, table legend, main text, or Methods section.

n/a	Confirmed	
☒	☐	The exact sample size (*n*) for each experimental group/condition, given as a discrete number and unit of measurement
☒	☐	A statement on whether measurements were taken from distinct samples or whether the same sample was measured repeatedly
☒	☐	The statistical test(s) used AND whether they are one- or two-sided<br>*Only common tests should be described solely by name; describe more complex techniques in the Methods section.*
☒	☐	A description of all covariates tested
☒	☐	A description of any assumptions or corrections, such as tests of normality and adjustment for multiple comparisons
☒	☐	A full description of the statistical parameters including central tendency (e.g. means) or other basic estimates (e.g. regression coefficient) AND variation (e.g. standard deviation) or associated estimates of uncertainty (e.g. confidence intervals)
☒	☐	For null hypothesis testing, the test statistic (e.g. *F*, *t*, *r*) with confidence intervals, effect sizes, degrees of freedom and *P* value noted<br>*Give P values as exact values whenever suitable.*
☒	☐	For Bayesian analysis, information on the choice of priors and Markov chain Monte Carlo settings
☒	☐	For hierarchical and complex designs, identification of the appropriate level for tests and full reporting of outcomes
☒	☐	Estimates of effect sizes (e.g. Cohen's *d*, Pearson's *r*), indicating how they were calculated

Our web collection on statistics for biologists contains articles on many of the points above.

Software and code

Policy information about availability of computer code

Data collection	The mapping analysis was done with Burrows-Wheeler Aligner (BWA, v0.7.17). FISH data were collected by a Leica epifluorescence microscope (DMI6000 B) together with Leica Application Suite X (3.4.2.18368.1.2). Genome assemblies were conducted using Canu (v1.8 for the M3 genome and v2.2 for the M5 genome), M3 assembly is error-corrected with Quiver (v2.2.1). Flye55 (v2.9.3) is used for aabys-male assembly, which is polished with Pilon56 (v1.24) using male illumina reads of the same strain.
Data analysis	Coverage analysis was done with SAMtools (v1.10). Summary statistics of the assembled M3 genome were obtained with QUAST51 (v4.6.3). BUSCO52 (v5.0.0) was used to estimate the completeness of assembled genomes. Repeat content of the M3 genome was analyzed with RepeatModeler (v1.0.11) and RepeatMasker (v4.0). Synteny between MV-contig and D. melanogaster was analyzed and plotted by R package RIdeogram. Homology search of sequences were done by BLAST (2.11.0+). Dotplot visualization of the alignments were done via Flexidot (v1.06). Coverage plot for different M loci was done with R package ggplot2.

For manuscripts utilizing custom algorithms or software that are central to the research but not yet described in published literature, software must be made available to editors and reviewers. We strongly encourage code deposition in a community repository (e.g. GitHub). See the Nature Portfolio guidelines for submitting code & software for further information.

Data

Policy information about availability of data

All manuscripts must include a data availability statement. This statement should provide the following information, where applicable:

- Accession codes, unique identifiers, or web links for publicly available datasets
- A description of any restrictions on data availability
- For clinical datasets or third party data, please ensure that the statement adheres to our policy

The authors affirm that all data necessary for supporting the findings are presented within the article, in figures, table, supplementary information and data or in the source data files. Genomic datasets produced in this study are available under BioProject: PRJNA1013067 and PRJNA1072234 in the NCBI database. Specifically, the accession numbers for those datasets are: genome M3 (JAVQME000000000), genome M5 (JAVVNY000000000), genome aabys-male (JAZGUT000000000), M2-male (Illumina reads, SRX21801162), M3-male_1 (Illumina reads, SRX21801164), M3-male_2 (Illumina reads, SRX21801165), M3-female_1 (Illumina reads, SRX21801166), M3-female_2 (Illumina reads, SRX21801167), M5-male (Illumina reads, SRX21801163). Accession numbers for applied previously-published datasets are: genome aabys (GCA_000371365.1), aabys-female (Illumina reads, SRX2154714), aabys-male (Illumina reads, SRX2154715), A3-female (Illumina reads, SRX2154716, A3-male (Illumina reads, SRX2154717), LPR-female (Illumina reads, SRX2154718), LPR-male (Illumina reads, SRX2154719). Accession numbers for the three reference gene used for Mdmd copy number analysis are Mdtra (GU070694.1), yellow (KY979110.1) and asense (XM_005176302.3).

Research involving human participants, their data, or biological material

Policy information about studies with human participants or human data. See also policy information about sex, gender (identity/presentation), and sexual orientation and race, ethnicity and racism.

Reporting on sex and gender	n/a
Reporting on race, ethnicity, or other socially relevant groupings	n/a
Population characteristics	n/a
Recruitment	n/a
Ethics oversight	n/a

Note that full information on the approval of the study protocol must also be provided in the manuscript.

Field-specific reporting

Please select the one below that is the best fit for your research. If you are not sure, read the appropriate sections before making your selection.

☒ Life sciences ☐ Behavioural & social sciences ☐ Ecological, evolutionary & environmental sciences

For a reference copy of the document with all sections, see nature.com/documents/nr-reporting-summary-flat.pdf

Life sciences study design

All studies must disclose on these points even when the disclosure is negative.

Sample size	Different numbers of samples were used in DNA extraction for sequencing, i.e., 5 adult individuals for each Illumina sequencing, 25 or 20 individuals for Pacbio RSII sequencing, 3 individuals for Pacbio HiFi sequencing. This was determined based on required DNA input by the sequencing facilities. For FISH experiments, 2-3 individuals were inspected for each strain as replicates. Signals considered as a successful hybridization were observed with consistent chromosomal locations on at least 20 metaphase nuclei.
Data exclusions	No data were excluded in this study.
Replication	Available replicates of genomic datasets, i.e. Illumina datasets of M3 males and females were analyzed separately to demonstrate reproducibility. Different analysis on the same datasets, i.e., Mdmd copy number analysis and M locus coverage analysis showed consistency in complexity of different M loci. For FISH experiments, signals were observed at least on 20 metaphase nuclei on a single slides and in 2-3 samples, demonstrating positive results. In case of M5 sample, no positive signals were observed 3 tested samples, showing undetectable MV locus using the applied protocol.
Randomization	Samples for genomic sequencing and experiments were randomly picked from housefly stocks, except for Dataset 1, 25 adult males were randomly picked from offsprings from a single pair of parents.
Blinding	Blinding was not relevant to this study as samples were specifically selected because of various male-determing loci that they possess.

Reporting for specific materials, systems and methods

We require information from authors about some types of materials, experimental systems and methods used in many studies. Here, indicate whether each material, system or method listed is relevant to your study. If you are not sure if a list item applies to your research, read the appropriate section before selecting a response.

Materials & experimental systems

n/a	Involved in the study	
☐	☒	Antibodies
☒	☐	Eukaryotic cell lines
☒	☐	Palaeontology and archaeology
☐	☒	Animals and other organisms
☒	☐	Clinical data
☒	☐	Dual use research of concern
☒	☐	Plants

Methods

n/a	Involved in the study	
☒	☐	ChIP-seq
☒	☐	Flow cytometry
☒	☐	MRI-based neuroimaging

Antibodies

Antibodies used	Probes for conducting FISH is labeled with digoxigenin(DIG)-11-deoxyuridine triphosphate (dUTP) and were detected with Anti-Digoxigenin-Rhodamine (Roche, Catalog number: 11207750910, LOT number: 51720500).
Validation	The antibody was commonly used in the field and validated by the manufacturer. See website below:<br>https://www.sigmaaldrich.com/NL/en/product/roche/11207750910<br>https://www.sigmaaldrich.com/NL/en/technical-documents/technical-article/genomics/nucleic-acid-labeling-and-detection/dig-labeling-methods

Animals and other research organisms

Policy information about studies involving animals; ARRIVE guidelines recommended for reporting animal research, and Sex and Gender in Research

Laboratory animals	Housefly: Strain M2, adult males were used for DNA extraction. Strain M3, adult males and females were used for DNA extraction; third instar larvae were used to prepare chromosome slides. Strain M5, adult males were used for DNA extraction; third instar larvae were used to prepare chromosome slides, pupae were used for verification of MdmdA and MdmdB transcription. Strain aabys: adult males were used for DNA extraction.
Wild animals	Italian, Spanish and Dutch housefly strains were originally collected from the field: strain ITA1 (originally collected in Altavilla Silentina, Italy), strain ITA3 (originally collected in Castelianeta marina, Italy), strain SPA1-5 (originally collected in Catalonia, Spain), strain NL1 (originally collected in Gerkesklooster, the Netherlands). Live flies were captured with net at local cattle farms and taken back to the laboratory and reared at 25°C with LD12:12h photoperiod in a climate room. Housefly stocks keep being maintained for research purposes.
Reporting on sex	The findings apply to houseflies that carry the male-determining loci, M, encoding the same primary signal gene, Mdmd. Tested individuals from strain M2, M3, M5, aabys, SPA3, SPA5 and NL1 are all male. Tested individuals from strain SPA1, SPA4, SPA5, ITA1 and ITA3 are untested for their biological gender as in those strains, a dominant female determiner traD exist, that can cause female development regardless the presence of M locus. This allows individuals carrying M locus to develop into female. As we are interested only in the M locus, we only tested our samples for M loci. Regarding detailed information of individuals and strains that we used, we refer to the following publication in addition to our current study.<br>Li, Xuan, et al. "Strong variation in frequencies of male and female determiners between neighboring housefly populations." Insect Science 29.5 (2022): 1470-1482.
Field-collected samples	This study did not use samples that are directly collected from the field.
Ethics oversight	The study was performed using insects that do not require ethical oversight in the Netherlands, where this study was performed in.

Note that full information on the approval of the study protocol must also be provided in the manuscript.

Plants

Seed stocks	n/a
Novel plant genotypes	n/a
Authentication	n/a
