# Supplementary material for: Divergent evolution of male-determining loci on proto-Y chromosomes of the housefly
Source: Nat Commun. 2024 Jul 16;15:5984. doi: 10.1038/s41467-024-50390-1 (PMC11252125; doi:10.1038/s41467-024-50390-1)
Supplement: Supplementary file 4 — Description of Additional Supplementary Files [file 41467_2024_50390_MOESM4_ESM.pdf]

Description of Additional Supplementary Files

File Name: Supplementary Data 1

Description: Identified *Mdmd*-like sequences in the M^{III} -contigs using the sequence of the complete *Mdmd* gene with Blast. The sequences are designated different *Mdmd* copies based on the sequence continuity on the contigs.

File Name: Supplementary Data 2

Description: Summary of repeats inserted within *Mdmd* copies in the M^{III} -contigs.
