# Supplementary figures and images for: Divergent evolution of male-determining loci on proto-Y chromosomes of the housefly
Source: Nat Commun. 2024 Jul 16;15:5984. doi: 10.1038/s41467-024-50390-1 (PMC11252125; doi:10.1038/s41467-024-50390-1)

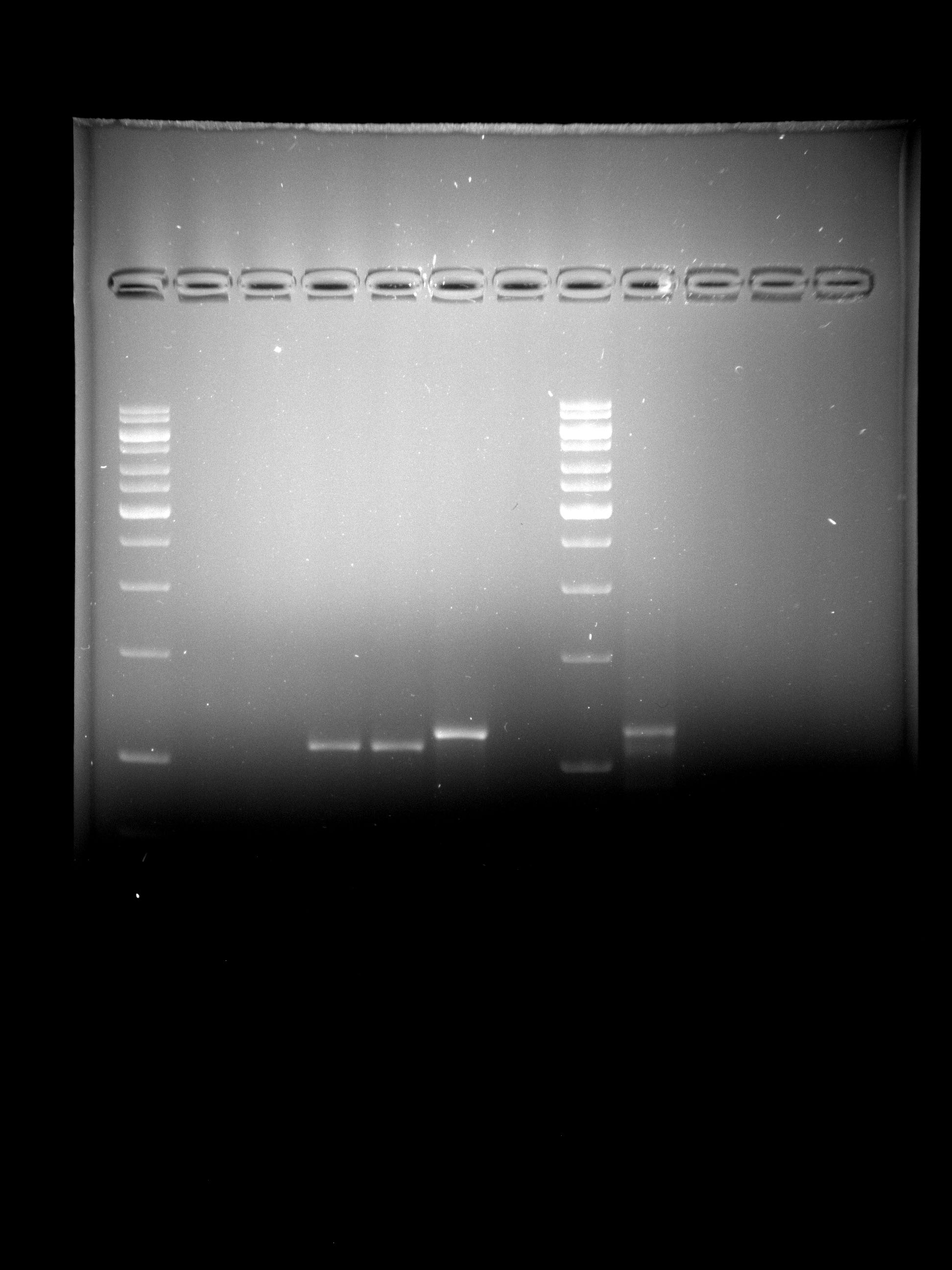

Supplement: Supplementary file 7 — Source data file [file 41467_2024_50390_MOESM7_ESM.zip › Uncropped scan of gel image-Supplementary fig. 2b.jpg]
